# Supplementary material for: Macroevolutionary changes in gene expression response to an immune stimulus across the diversity of fishes
Source: Mol Biol Evol. 2025 Dec 15;43(1):msaf323. doi: 10.1093/molbev/msaf323 (PMC12839524; doi:10.1093/molbev/msaf323)
Supplement: msaf323_Supplementary_Data [file msaf323_supplementary_data.zip › Multispecies DiffExpr_Nov21_Supp_Figs.pdf]

Supplemental Figures:

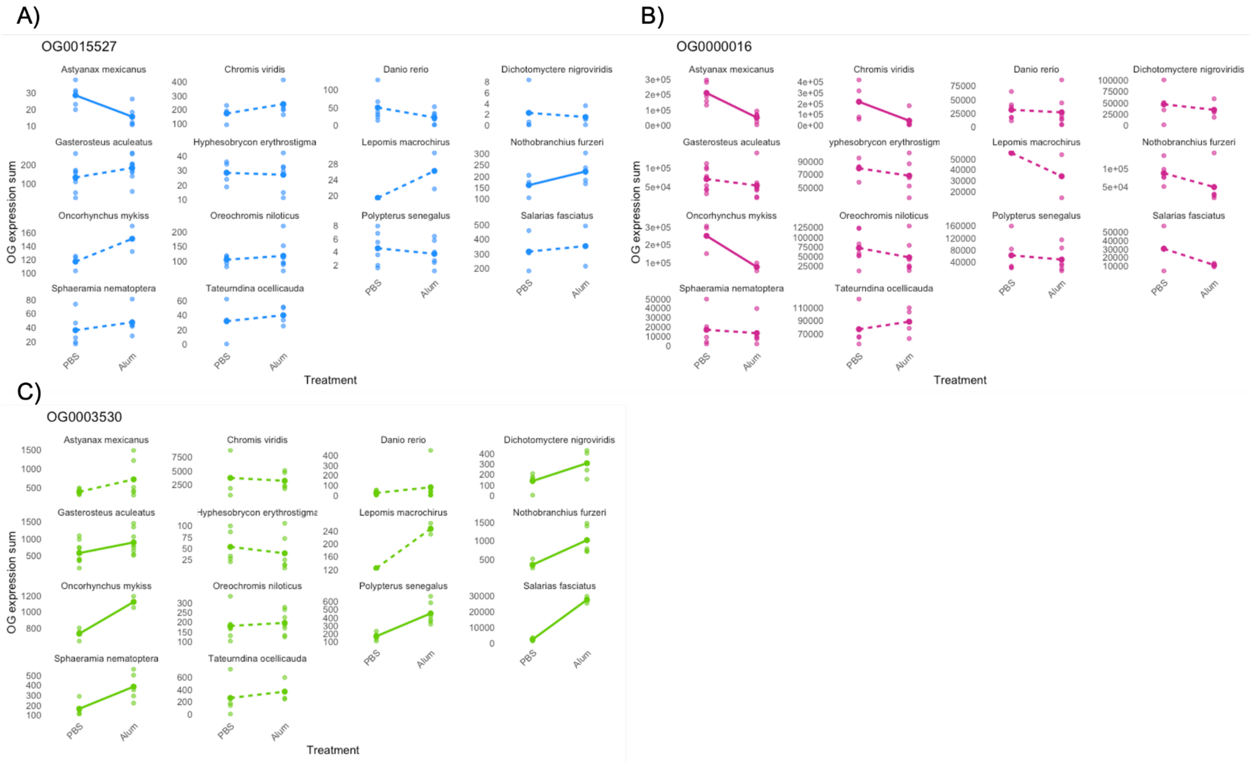

Figure S1: Orthogroup expression for PBS and Alum injections across all species where solid lines represent a significant treatment effect (p-value < 0.1) and dotted line represents non-significant treatment effect (p-value > 0.1) for A) a single-copy ortholog, B) orthogroup with highest effect size of treatment (OG0000016), and C) orthogroup with highest effect size of the interaction of treatment and species (OG0003530).

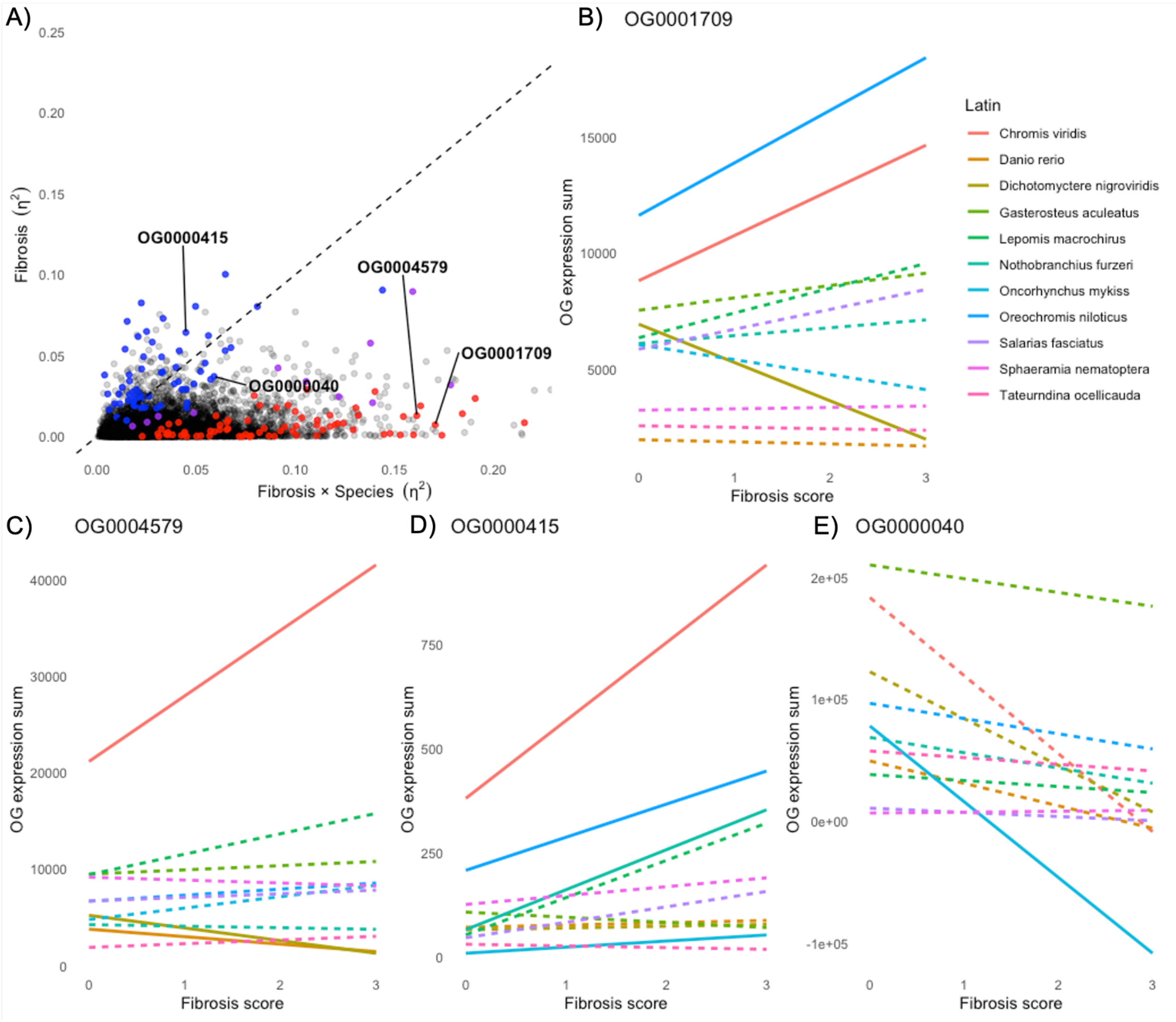

Figure S2: A) For each orthogroup summed normalized gene expression, we used a linear model to estimate the effect size (η^2) for the effects of fibrosis score, and the fibrosis score × species interaction on variation in the orthogroup expression . Treatment η^2 indicates the extent to which any orthogroup summed normalized expression diverges predictably for fibrosis. The η^2 for the fibrosis × species interaction measures the extent to which fibrosis depends on the species for every orthogroup. The dashed line is a 1:1 line, for ease of visualization; points falling above this line have a larger fibrosis effect than interaction effect with blue significant

effect of fibrosis only, purple fibrosis and interaction, and red only the interaction term (p-value < 0.1). Orthogroup summed expression for B) OG0000415, C) OG0000040, D) OG0004579), and E) OG0001709 where solid lines represent a significant fibrosis effect (p-value < 0.1).
